# Alterations in 18F-FDG accumulation into neck-related muscles after neck dissection for patients with oral cancers

**DOI:** 10.4317/medoral.21018

**Published:** 2016-03-31

**Authors:** Shinji Kito, Hirofumi Koga, Masaaki Kodama, Manabu Habu, Shinya Kokuryo, Masafumi Oda, Kou Matsuo, Takanobu Nishino, Shinobu Matsumoto-Takeda, Masataka Uehara, Daigo Yoshiga, Tatsurou Tanaka, Shun Nishimura, Ikuya Miyamoto, Masaaki Sasaguri, Kazuhiro Tominaga, Izumi Yoshioka, Yasuhiro Morimoto

**Affiliations:** 1 DDS PhD. DDS. Division of Oral and Maxillofacial Radiology, Kyushu Dental University, Kitakyushu, JAPAN; 2 MD PhD. Kitakyushu PET Center, Nishinippon Sangyoeiseikai, Kitakyushu, JAPAN; 3 DDS PhD. Division of Maxillofacial Surgery, Kyushu Dental University, Kitakyushu, JAPAN; 4 DDS PhD. Division of Oral Medicine, Kyushu Dental University, Kitakyushu, JAPAN; 5 DDS PhD. Division of Oral Pathology, Kyushu Dental University, Kitakyushu, JAPAN; 6 DDS PhD. Division of Comprehensive Dentistry, Kyushu Dental University, Kitakyushu, JAPAN; 7 DDS PhD. Center for Oral Biological Research, Kyushu Dental University, Kitakyushu, JAPAN

## Abstract

**Background:**

18F-fluoro-2-deoxy-D-glucose (18F-FDG) accumulations are commonly seen in the neck-related muscles of the surgical and non-surgical sides after surgery with neck dissection (ND) for oral cancers, which leads to radiologists having difficulty in diagnosing the lesions. To examine the alterations in 18F-FDG accumulation in neck-related muscles of patients after ND for oral cancer.

**Material and Methods:**

18F-FDG accumulations on positron emission tomography (PET)-computed tomography (CT) in neck-related muscles were retrospectively analyzed after surgical dissection of cervical lymph nodes in oral cancers.

**Results:**

According to the extent of ND of cervical lymph nodes, the rate of patients with 18F-FDG-PET-positive areas increased in the trapezius, sternocleidomastoid, and posterior neck muscles of the surgical and/or non-surgical sides. In addition, SUVmax of 18F-FDG-PET-positive areas in the trapezius and sternocleidomastoid muscles were increased according to the extent of the ND.

**Conclusions:**

In evaluating 18F-FDG accumulations after ND for oral cancers, we should pay attention to the 18F-FDG distributions in neck-related muscles including the non-surgical side as false-positive findings.

**Key words:**18F-FDG, PET-CT, oral cancers, muscles.

## Introduction

Surgical dissections are usually performed for patients with oral cancers and metastatic lymph nodes. Since the procedures induce anatomical and histological injuries of the tissues, precise evaluation for the recurrence of primary cancers and metastatic tumors of lymph nodes becomes very difficult using only computed tomography (CT) and magnetic resonance imaging (MRI) (1,2). Therefore, positron emission tomography (PET)-CT using fluorine-18-labeled (18F) fluoro-2-deoxy-D-glucose (18F-FDG) should be used to evaluate recurrence of oral cancers and metastatic lymph nodes, and the clinical applications of this modality have expanded widely (1-6).

However, in our experience, 18F-FDG accumulations are commonly seen in the neck-related muscles of the surgical and non-surgical sides after surgery with neck dissection (ND) for oral cancers, which leads to radiologists having difficulty in diagnosing the lesions. To the best of our knowledge, there have been no reports on 18F-FDG distribution in neck-related muscles of the oral and maxillofacial regions after surgery with ND for oral cancer.

In the present study, the alterations in the 18F-FDG distributions in neck-related muscles of the oral and maxillofacial regions before and after surgery with ND for oral cancer were analyzed.

## Material and Methods

Eighty consecutive patients (49 male, 31 female; mean ± standard deviation (SD) age 65.2±10.4 years, age range 22-93 years) who had undergone surgery for oral cancers were evaluated at Kyushu Dental University hospital between 2005 and 2014 ( Table 1). CT, MRI, and 18F-FDG-PET-CT were performed in all 80 patients. Informed consent was obtained before the respective imaging examinations. All procedures followed were in accordance with the ethical standards of the responsible committee on human experimentation and with the Helsinki Declaration. Approval of the present study was obtained from the institutional review board of Kyushu Dental University (No. 12-13).

**Table 1:**
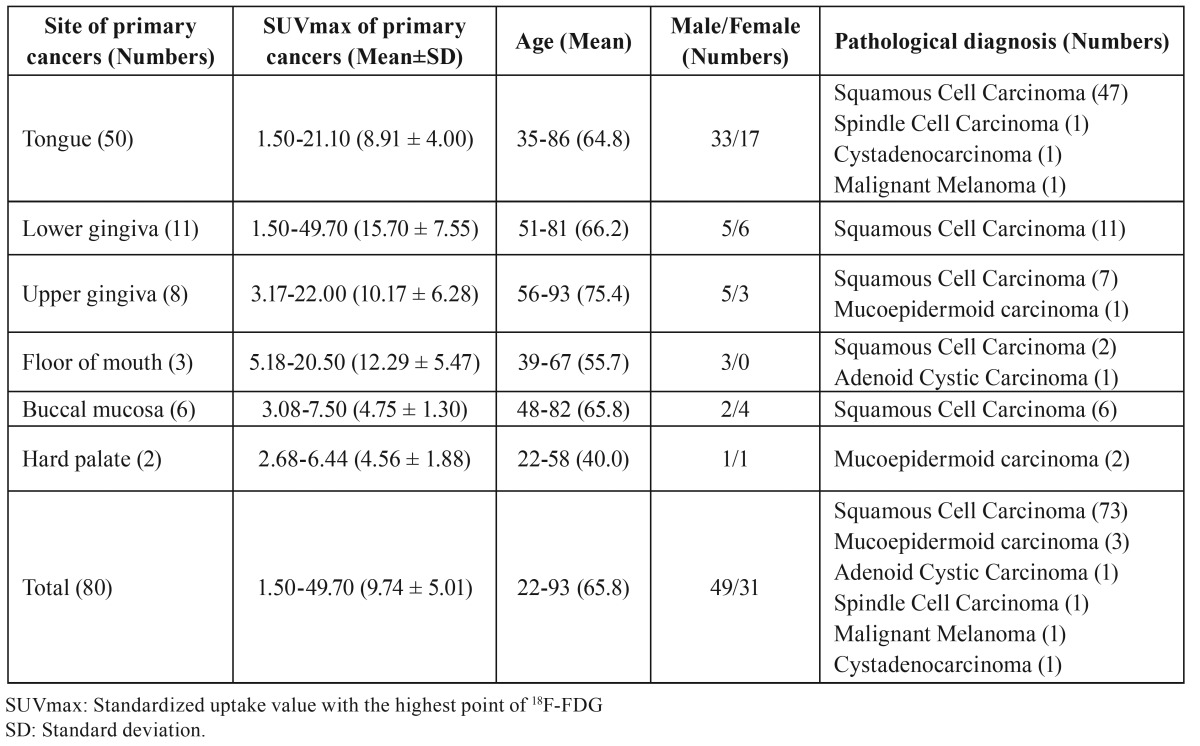
Data summary in 80 patients with oral cancers.

Eighty patients were retrospectively divided into 4 groups in the present study according to the extent of ND: Group 1 included 25 patients without ND; Group 2 included 16 patients with supraomohyoid ND (SOHND) on the same side as the primary tumor, including 2 bilateral cases; Group 3 included 24 patients with radical ND (RND) on the same side as the primary tumor; and Group 4 included 15 patients with RND on the same side as the primary tumor plus SOHND on the side contralateral to the primary tumor. CT, MRI, and 18F-FDG-PET-CT were performed at least six months after surgery, because postoperative inflammation has generally almost completely disappeared by then. All imaging acquisitions were done within one month. Patients whose CT, MRI, and 18F-FDG-PET-CT examinations were not all performed within one month were excluded. If all were to be performed, 18F-FDG-PET-CT could not be performed at the first visit to a doctor and/or within six months after the surgery.

All patients fasted for at least 4 hours prior to intravenous administration of 0.1 mCi (3.7 MBq)/kg body weight of 18F-FDG. The examination by 18F-FDG was performed with a PET-CT inline system (Discover ST Elite, GE Medical Systems, Waukesha, WI, USA). One hour after 18F-FDG injection, each patient underwent a single integrated PET-CT examination. Patients were instructed not to chew or talk during the study. Prior to scanning, all patients had to remove objects such as removable dental bridgework and dentures from their mouth. The patients were positioned in the head-first, supine position. They were instructed to perform breath holding during the CT acquisition, which was done first from the head to the pelvic floor. For PET-CT imaging, the following parameters for the CT scan were used: 140 kV, 60-160 mA (2D Auto mA), 0.6 seconds per tube rotation, slice thickness of 4.25 mm, helical pitch of 1.5:1, 22.50 mm/rotation table speed, 824.5-1096.5 mm coverage, and 22.8-30.0 seconds acquisition time. Immediately following the CT scan, a PET scan was acquired from the pelvic floor to the head. The PET camera had 18 rings of 336 detectors and simultaneously produced 35 4.34-mm-thick slices, along a 152-mm axial field of view (FOV), with 3-slice overlap at the borders of the FOV. The trans-axial resolution was 4.8 mm with full width at half-maximum at 1 cm radius from the center, and the sensitivity of the device was 1.3 cps/kBq with 2D acquisition time of 2 minutes, resulting in a total scanning time of 18-24 minutes with each scan.

The CT data were back-projected to the workstation; the images were degraded from a 512 x 512 matrix to a 128 x 128 matrix size to correspond with the PET images, and they were also forward-projected. The resulting sinograms were filtered with a 6.00-mm Post filter or a 4.30-mm Loop filter, exponentiated, and entered as attenuation correction factors into the PET image reconstruction with two iterations and 21 subsets. On the other hand, all PET studies were iteratively reconstructed with no attenuation correction.

MR images were acquired using a 1.5-T full-body MR system (EXCELART VantageTM Powered by Atlas; Toshiba Co. Ltd., Tokyo, Japan) with a circular polarized neck coil to visualize the maxilla and mandible level. Enhanced-CT was performed with an Aquilion™ machine (Toshiba Co. Ltd.) after the patient received an intravenous dose of 50 mL iohexol (300 mgI/mL; Omnipaque 300™, Daiichi Pharmaceutical Co. Ltd., Tokyo, Japan) at the start of scanning and an additional 50-mL intravenous infusion during the scanning to allow better visualization of the vascular structures. Scanning was performed in the axial plane without angulation, in 5-mm-thick contiguous sections from the cavernous sinuses to the thoracic inlet.

The PET-CT, MR, and CT images were independently assessed by two expert radiologists (K.S. and K.H.).

All PET images were analyzed on a PC-based, digital viewing system (GE view, Dornstadt, Germany) to choose transverse slices for comparison and to adapt the level of the gray scale in the images. The extents of 18F-FDG accumulations were judged by the standardized uptake value of the highest point within the regions of interest (SUVmax) for the representative neck-related muscles before the treatments and after surgery. The masseters, the anterior bellies of the digastric muscles, the geniohyoid, trapezius, sternocleidomastoid muscles, and posterior neck muscles together with the spinalis cervicis muscles, semispinalis cervicis muscles, semispinalis capitis muscles, and splenius capitis muscles were investigated as the neck-related muscles. An SUVmax cutoff of 1.5 was chosen as the presence or the absence of 18F-FDG accumulations, because it is commonly used as the no-color area in a 4-grade system, and the area could be easily judged (7). In addition, radiologists generally pay no attention to such areas. In addition, the precise SUVmax of 18F-FDG for 18F-FDG-PET-positive areas was measured in the respective muscles mentioned above on PET-CT images.

All statistical analyses were performed using Stat View™ version 5.0 software (SAS Institute Inc., Cary, NC, USA). Differences in mean values among groups were analyzed using the Kruskal-Wallis test. Correlations between two categories were analyzed using Spearman’s correlation coefficient. Results were considered significant if *p*<0.05.

## Results

- Distribution of sites and SUVmax in primary sites of oral cancers

The distribution of the sites and of the SUVmax of the 18F-FDG accumulations in the primary sites of oral cancers in the 80 patients are shown in  Table 1. The most common site was the tongue (50). The major pathological diagnosis was squamous cell carcinoma (SCC). The SUVmax of 18F-FDG in the primary lesions ranged from 1.50 to 49.70 (mean ± standard deviation (SD): 9.74 ± 5.00) ( Table 1).

18F-FDG accumulations in neck-related muscles before surgery for patients with oral cancers

18F-FDG accumulations in neck-related muscles before surgery for patients with oral cancers are shown in  Table 2. In all Groups, there were almost no patients with 18F-FDG-positive areas in all examined muscles before surgery, and no significant differences were found in the rates of patients with 18F-FDG-positive areas among 6 muscles in the 4 Groups ( Table 2). Of course, no significant differences were found in the rates of patients with 18F-FDG-positive areas among the 4 Groups in the respective 6 muscles ( Table 2).

**Table 2:**
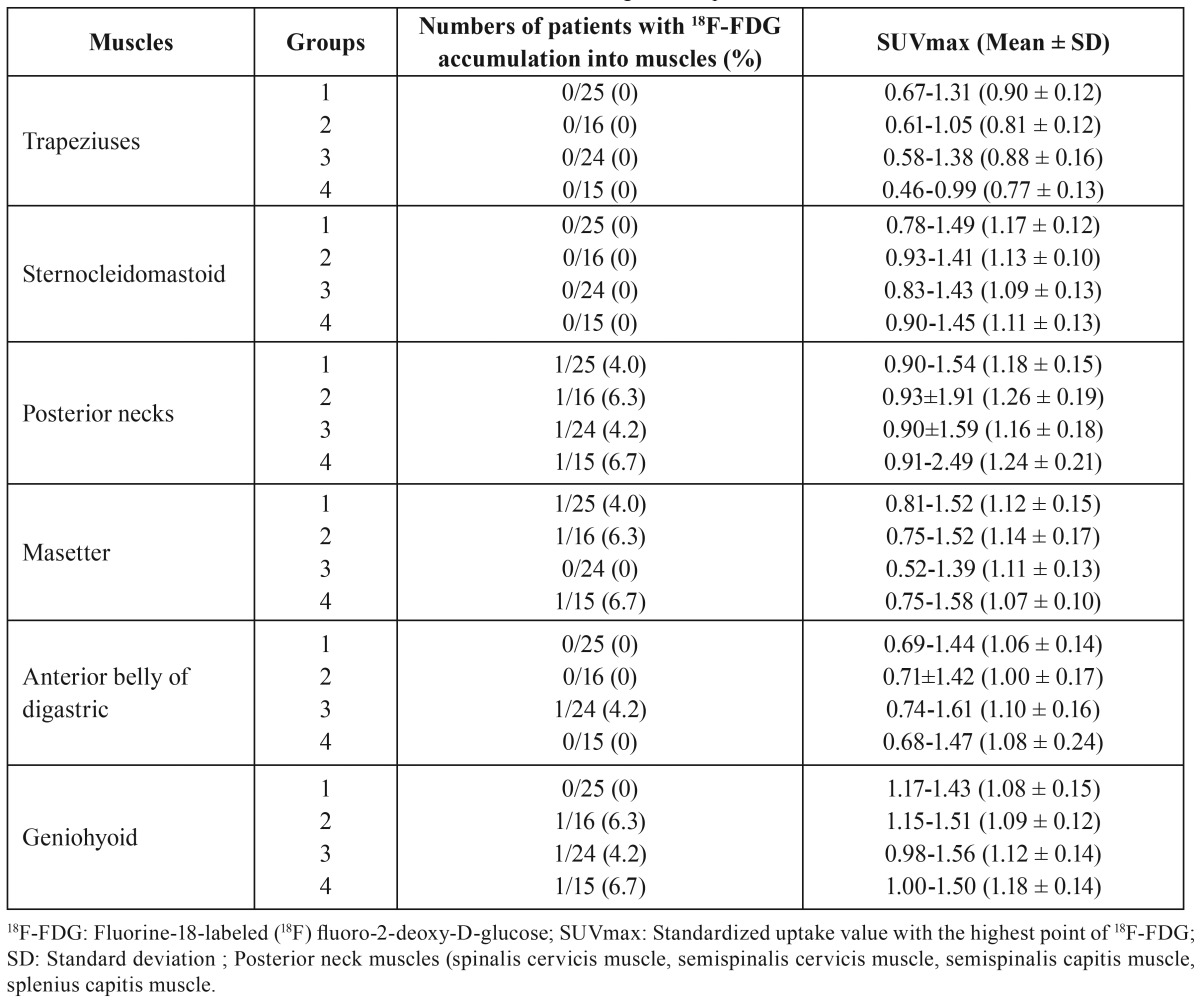
18F-FDG accumulations into neck-related muscles before surgeries for patients with oral cancers.

18F-FDG accumulations in neck-related muscles after minimally invasive surgery (Groups 1 and 2) for patients with oral cancers.

**Figure 1:**
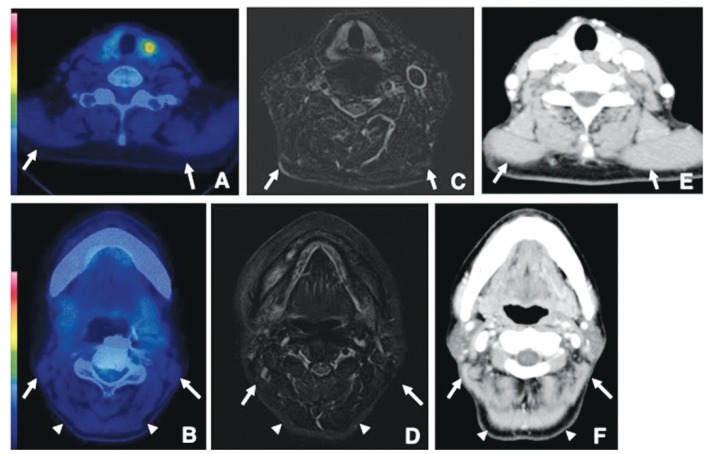
18F-FDG-PET-CT, CT, and MRI examinations of a 61-year-old woman 8 months after surgery without ND for SCC of the right side of the gingiva. 18F-FDG-PET-CT demonstrates no increased uptake in the trapezius (arrows) (A), sternocleidomastoid (arrows), and posterior neck muscles (arrowheads) (B). STIR shows no signal changes of the trapezius (arrows), (C) sternocleidomastoid (arrows), and posterior neck muscles (arrowheads) (D). CT with a soft tissue window shows no density changes of the trapezius (arrows) (E), sternocleidomastoid (arrows), and posterior neck muscles (arrowheads) (F).

No significant differences were found in the rates of patients with 18F-FDG-PET-positive areas in the trapezius, sternocleidomastoid, and posterior neck muscles between before and after surgery in Groups 1 and 2 ( Table 2, Table 3). In Groups 1 and 2, no significant differences were found in the rates of patients with 18F-FDG-positive areas among the 6 muscles after surgery (Fig. 1 and  Table 3).

**Table 3:**
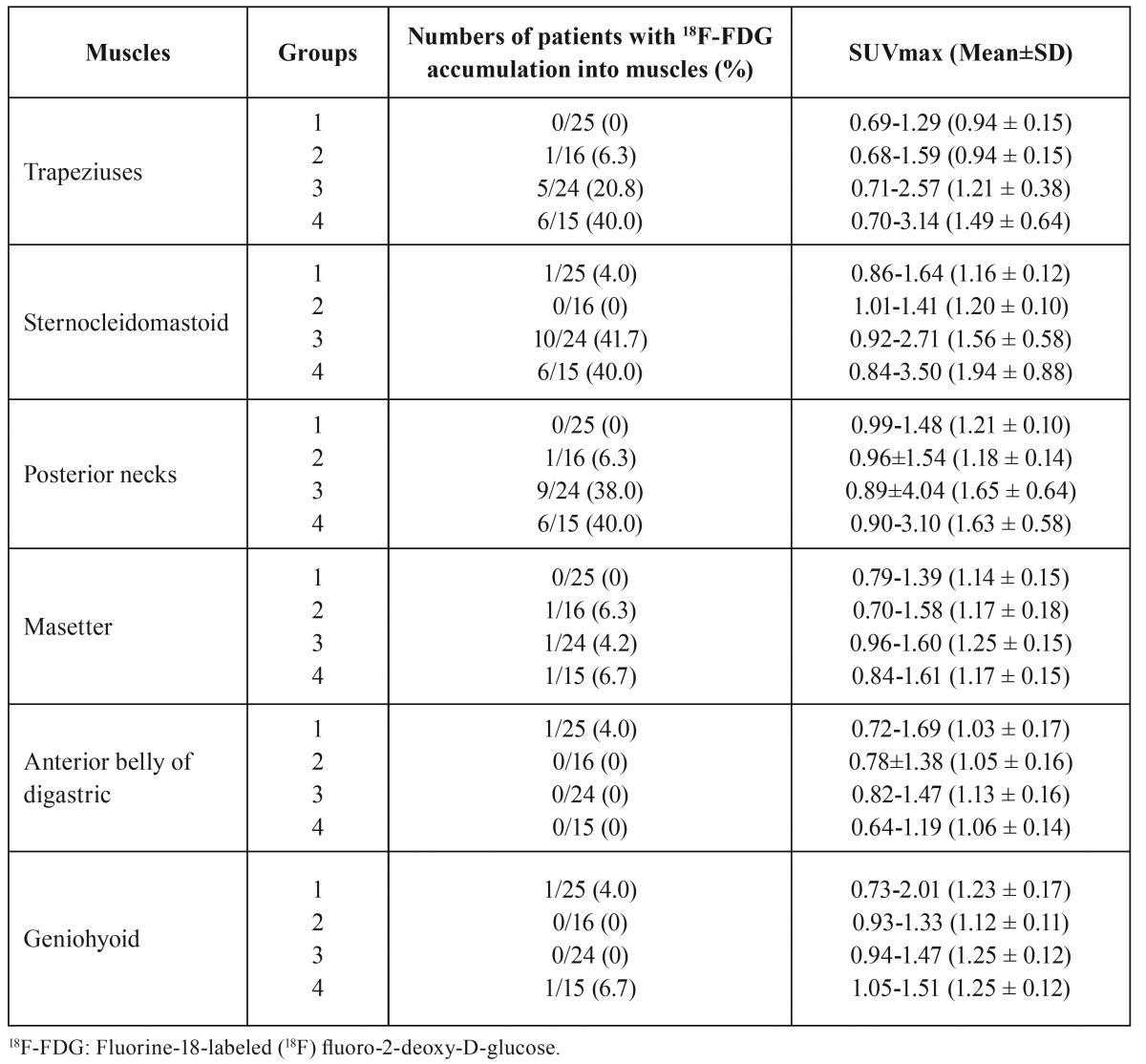
18F-FDG accumulations into neck-related muscles after surgeries for patients with oral cancers.

Alterations in 18F-FDG accumulations in neck-related muscles after invasive surgery (Groups 3 and 4) for patients with oral cancers.

**Figure 2:**
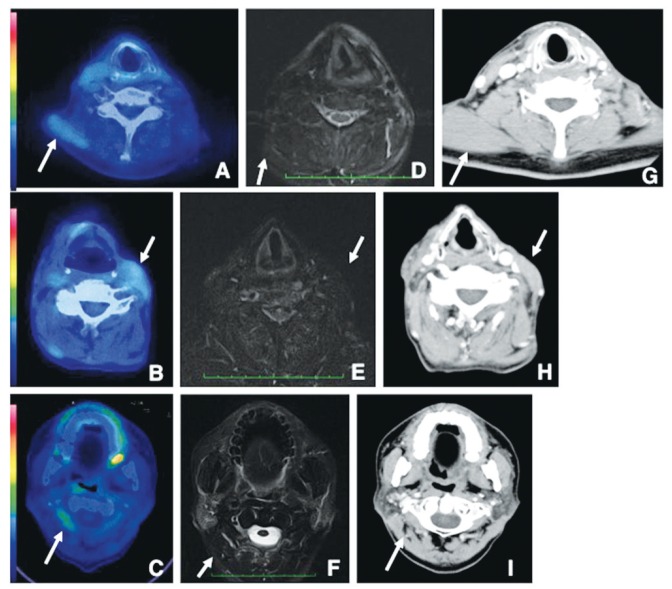
18F-FDG-PET-CT, CT, and MRI examinations of a 51-year-old man 18 months after surgery with ND for SCC of the left side of the mandibular gingiva (A, D, G); of a 78-year-old man 9 months after surgery with ND for SCC on the right side of the tongue (B, E, H); and of a 61-year-old man 35 months after surgery with ND for SCC on the right side of the tongue (C, F, I). 18F-FDG-PET-CT demonstrates increased uptake in the trapezius (SUVmax 2.1) (arrows) (A), sternocleidomastoid (SUVmax 1.9) (arrows) (B), and posterior neck muscles (SUVmax 2.2) (arrows) (C). STIR shows no signal changes of the trapezius (arrows) (D), sternocleidomastoid (arrows) (E), and posterior neck muscles (arrows) (F) in 18F-FDG-PET-positive areas. CT with a soft tissue window shows no density changes of the trapezius (arrows) (G), sternocleidomastoid (arrows) (H), and posterior neck muscles (arrows) (I) in 18F-FDG-PET-positive areas.

In Group 3, the rates of patients with 18F-FDG-PET-positive areas were significantly increased in the trapezius (*p*<0.004), sternocleidomastoid muscles of the non-surgical side (*p*<0.0001), and posterior neck muscles of the non-surgical side (*p*<0.0001) between before and after surgery (Fig. 2 and  Table 3), with similar results in Group 4 (Fig. 2 and  Table 3). On the other hand, no significant differences were found in the rates of patients with 18F-FDG-PET-positive areas in bilateral masseters, bilateral anterior bellies of the digastric muscles, and bilateral geniohyoid muscles between before and after surgery ( Table 2, Table 3). There were no abnormal findings in bilateral trapezius muscles, sternocleidomastoid muscles of the non-surgical side, and posterior neck muscles of the non-surgical side in patients with 18F-FDG-PET-positive areas on CT and MRI (Fig. 2).

Correlations between the alterations in 18F-FDG accumulations in neck-related muscles after surgery and the extent of dissection in patients with oral cancers

A moderate correlation was found between the rates of patients with 18F-FDG-PET-positive areas in the trapezius muscles and the extent of surgical dissection in the 4 Groups (r=0.40, *p*<0.0001), with moderate correlations for the sternocleidomastoid muscles (r=0.442, *p*<0.0001) and the posterior neck muscles (r=0.445, *p*<0.0001). Moreover, a strong correlation was found between the rates of patients with 18F-FDG-PET-positive areas in at least one of the trapezius, sternocleidomastoid, and posterior neck muscles and the extent of surgical dissection in the 4 Groups (r=0.665, *p*<0.01) ( Table 4).

**Table 4:**
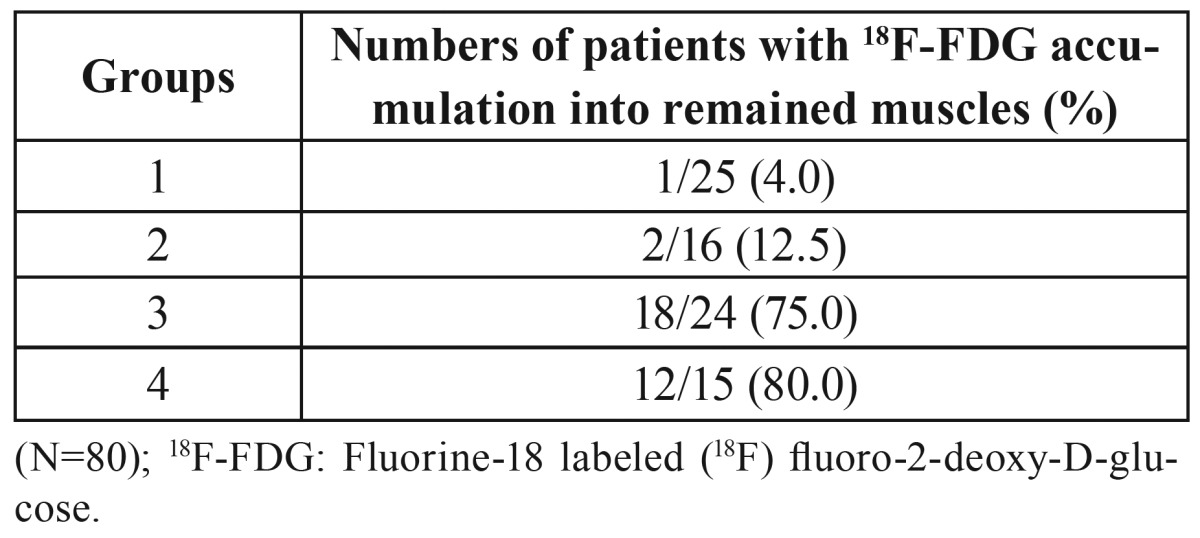
Relationships between the rates of patients with 18F-FDG-PET-positive areas and the extent of surgical methods.

The SUVmax values of 18F-FDG-PET-positive areas in the trapezius, sternocleidomastoid, and posterior neck muscles after surgery ranged from 1.50 to 4.04 ( Table 3). In addition, a weak correlation was found between the SUVmax of 18F-FDG-PET-positive areas in the trapezius muscles and the extent of surgical dissection in the 4 Groups (r=0.32, *p*<0.002), as well as a weak correlation in the sternocleidomastoid muscles (r=0.258, *p*<0.011).

## Discussion

The most interesting result of the present study was that 18F-FDG-PET-positive areas appeared in the trapezius, sternocleidomastoid, and posterior neck muscles of the surgical and non-surgical sides after RND for oral cancers despite no abnormal findings, including hypertrophy, on CT and MRI. In addition, the rates of patients with 18F-FDG-PET-positive areas increased depending on the extent of the dissection for cervical lymph nodes. There have been no previous reports on 18F-FDG distribution in neck-related muscles of the oral and maxillofacial regions after surgery with ND for oral cancer. It has been reported that hypertrophy of the levator scapulae muscles, trapezius muscles, and sternocleidomastoid muscles occurred on the non-surgical side after RND of the neck using electromyography in previous reports (8-11). In addition, it was noted that radiologists should pay attention to the differential diagnosis between tumors and hyperplasia of the muscles mentioned above on CT and MRI examinations (12). Based on the present results, however, it is important to understand that possible increases of 18F-FDG accumulations in the muscles mentioned above are unrelated to hypertrophy when they show 18F-FDG accumulation after RND.

Moreover, SUVmax values of 18F-FDG-PET-positive areas of the trapezius, sternocleidomastoid, and posterior neck muscles after surgery were relatively low, but ranged from 1.5 to 4.04. In previous studies, as well as in the present study (1.50 to 49.70), the SUVmax of carcinomas in the oral cavity ranged from 1.38 to 15.9; there is some overlap in the SUVmax caused by dental inflammation and that caused by carcinomas (7,13-16). It is important to perform an appropriate differential diagnosis between normal variations after surgery and malignant tumors on CT and/or MRI, in addition to paying attention to alterations in the distribution of 18F-FDG accumulations. In particular, it is important to pay attention to differentiating between the two, since SUVmax values of 18F-FDG accumulations tend to increase with extensive surgical methods such as RND.

It is difficult to explain the present results, since this was the first study of 18F-FDG distribution after surgery for oral and maxillofacial cancers. One of the possible explanations for these findings is that the 18F-FDG-PET-positive areas of the trapezius, sternocleidomastoid muscles, and posterior neck muscles may be related to the muscles’ hypertonia and the compensation that occurs with neck exercise (8-11). The other is that stiff-neck after surgeries could be related to muscles with 18F-FDG-PET-positive areas. Further study is needed to elucidate the reasons behind these results.

One possible limitation of the present study is that the sample size was small. The variables of age, race, and sex could not be studied in this study sample. In addition, the analyzed data did not include the precise dissection areas and the methods and extent of reconstruction. Therefore, the present results should be considered with care. However, we should pay attention to alterations in the distributions in adjacent remaining muscles, including non-surgical sides, when evaluating 18F-FDG accumulations after surgery for oral cancer.
